# Donor-derived Cell-free DNA for Detection of Rejection After Pancreas Transplantation

**DOI:** 10.1097/TXD.0000000000001939

**Published:** 2026-04-08

**Authors:** Abraham J. Matar, Samy Riad, Heidi Sarumi, Michael G. Megaly, Matthew Wright, Erik Finger, Vanessa Humphreville, Karthik Ramanathan, Jessica Fisher, Joseph S. Rao, Zachary Demko, Adam Prewett, Hossein Tabriziani, Raja Kandaswamy

**Affiliations:** 1 Division of Transplantation, Department of Surgery, University of Minnesota, Minneapolis, MN.; 2 Division of Nephrology, Department of Medicine, Mayo Clinic, Rochester, MN.; 3 Natera, Inc, San Carlos, CA.

## Abstract

**Background.:**

Donor-derived cell-free DNA (dd-cfDNA) has emerged as a promising noninvasive biomarker for detecting allograft rejection in solid organ transplantation. However, its diagnostic utility in pancreas transplantation, including in multiorgan recipients, remains underexplored.

**Methods.:**

We conducted a retrospective analysis of 42 pancreas transplant recipients (38 simultaneous pancreas-kidney, 4 pancreas transplant alone) who underwent for-cause organ biopsy and dd-cfDNA testing between April 2020 and December 2024. dd-cfDNA was measured using the Prospera assay, with a threshold of ≥1% considered high risk and <1% low-risk. Biopsy results were correlated with dd-cfDNA to assess test performance.

**Results.:**

A total of 50 dd-cfDNA tests and 45 organ biopsies were analyzed. Among simultaneous pancreas-kidney recipients, dd-cfDNA demonstrated a sensitivity of 86.7%, specificity of 52.2%, positive predictive value (PPV) of 54.2%, and negative predictive value (NPV) of 85.7% for rejection. In pancreas transplant alone recipients, all high-risk dd-cfDNA results corresponded to biopsy-proven rejection, yielding 100% sensitivity, specificity, PPV, and NPV. When combining both cohorts, dd-cfDNA testing achieved an overall sensitivity of 88.2%, specificity of 57.7%, PPV of 57.7%, and NPV of 88.2%. Additionally, dd-cfDNA kinetics suggested its potential utility as a surveillance tool posttreatment, potentially reducing the need for closure biopsies.

**Conclusions.:**

dd-cfDNA is a valuable noninvasive adjunct for monitoring of rejection in pancreas transplant recipients. Further prospective studies are needed to validate these findings and define the optimal role of dd-cfDNA in posttransplant care.

## INTRODUCTION

Outcomes following pancreas transplantation have significantly improved over time, owing in part to advances in diagnostic techniques that facilitate earlier and more precise detection of graft rejections.^1-3^ Although clinical symptoms and laboratory markers—such as serum amylase and lipase—may raise suspicion for rejection, these indicators lack adequate sensitivity and specificity. When feasible, percutaneous pancreas biopsy is the gold standard for diagnosis of pancreas graft rejection.^4^ Percutaneous pancreas biopsy allows for direct histological assessment, enabling accurate diagnosis, grading of rejection severity, and differentiation between antibody-mediated and cellular rejection.^5^ Moreover, percutaneous biopsy enables clinicians to differentiate rejection from other causes of graft dysfunction, including pancreatitis or infection, allowing for more targeted and effective treatment strategies.

Despite the utility of percutaneous pancreas biopsy for diagnosis of graft rejection, it is not without drawbacks.^5^ The procedure carries risks such as bleeding, pancreatic fistula, inadvertent injury to adjacent organs and, rarely, major hemorrhage that may require surgical intervention.^6,7^ The biopsy failure rate can be up to 30%, particularly in the presence of peripancreatic fluid or graft pancreatitis, and the diagnostic utility is highly dependent on the experience of the operator and pathologist.^5^ Additionally, technical challenges may arise in cases of small or poorly accessible grafts, such as in the case of overlying intestines precluding safe percutaneous access. In the case of simultaneous pancreas-kidney (SPK) transplantation, some advocate for biopsy of the kidney as a surrogate when a pancreas biopsy cannot be obtained because of anatomical feasibility or other reasons. However, recent reports suggest poor concordance of kidney and pancreas biopsies in SPK recipients, rendering this practice unreliable.^8-10^

Donor-derived cell-free DNA (dd-cfDNA) has emerged as a promising noninvasive biomarker for detecting allograft rejection in solid organ transplantation including kidney, liver heart, and lung transplantation.^11-14^ However, its application in pancreas transplantation—including multiorgan recipients—remains underexplored.^15,16^ Existing studies in pancreas transplant populations have primarily focused on posttransplant dd-cfDNA trends and their correlation with protocol pancreas biopsy results. Significant knowledge gaps persist regarding dd-cfDNA’s diagnostic performance, including its sensitivity and specificity for detection of rejection, as well as its interpretative challenges in recipients with concurrent organ grafts. The objective of this study was to evaluate the diagnostic accuracy of dd-cfDNA for the detection of rejection in pancreas transplant recipients undergoing for-cause biopsy.

## MATERIALS AND METHODS

### Study Population

This retrospective study includes all recipients of pancreas transplantation—including multiorgan transplant recipients—who underwent for-cause biopsy between April 2020 and December 2024 at the University of Minnesota and had dd-cfDNA testing performed contemporaneously with the biopsy. During this period, our transplant program was following an estimated 500 pancreas transplant recipients. Of these, 42 patients with both biopsy and dd-cfDNA testing were included in the current analysis. During this investigational study period, there was no standardized protocol for the timing of dd-cfDNA testing and biopsy. dd-cfDNA was typically ordered at the first sign of potential immune-mediated injury, while biopsy timing was based on clinical discretion, leading to variability in intervals between tests in this series. Indications for biopsy were also heterogeneous and included biochemical evidence of graft dysfunction (eg, elevations in lipase and/or creatinine above patient baseline), fever of unknown origin, development of de novo donor-specific antibodies, and/or subtherapeutic immunosuppression levels (tacrolimus levels <5 ng/mL). All biopsies received by patients in this cohort were “for cause,” rather than “protocol biopsies.” This study received approval by the University of Minnesota Institutional Review Board (STUDY00021020). The need for written informed consent was waived by the Institutional Review Board.

### Donor-derived Cell-free DNA Measurement

Blood samples were sent to an external laboratory for dd-cfDNA testing using the Prospera Test (Natera, Austin, TX), and the proportion of dd-cfDNA was reported. The Prospera Test is a noninvasive test that estimates the fraction of dd-cfDNA by measuring allele frequencies at >13 000 single nucleotide polymorphisms selected to maximize informativeness across diverse ethnicities. Cell-free DNA extraction, library preparation, and amplification were performed, followed by target enrichment using massively multiplexed polymerase chain reaction targeting these single nucleotide polymorphisms, as previously described.^16^ Sequencing was conducted on an Illumina HiSeq 2500 Rapid Run platform, generating 10–11 million reads per sample. In accordance with clinical validation studies of this assay in SPK patients, a threshold of ≥1% dd-cfDNA was used to distinguish high-risk (positive) from low-risk (negative) results.^17^ While a threshold has not yet been established for pancreas transplant alone (PTA) patients, we used the same threshold for exploratory purposes.

### Immunosuppression

At the University of Minnesota, rabbit antithymocyte globulin induction is the primary induction agent for pancreas transplantation.^1^ SPK recipients receive a total of 6 mg/kg rabbit antithymocyte globulin administered in 4 divided doses, while solitary pancreas recipients receive a total of 7.5 mg/kg in 5 divided doses. Maintenance immunosuppression consists of tacrolimus and mycophenolate, with early steroid withdrawal implemented within 5 d posttransplant. All pancreas transplants were crossmatch negative and ABO compatible.

### Pancreas Transplantation

Pancreas transplantation was performed as previously described.^18^ Briefly, a midline exploratory laparotomy is made, and the pancreatic graft is placed intraperitoneally in the right lower quadrant of the pelvis. This intraperitoneal approach facilitates vascular anastomosis to the iliac vessels and enteric drainage by duodenoenterostomy. The kidney graft is also placed intraperitoneally in the left iliac fossa.

### Biopsies

Pancreas biopsies were performed under computed tomography guidance. Using sterile technique, a needle introducer was advanced to the tail of the pancreas, and tissue cores were obtained with a spring-loaded 18-gauge biopsy gun. Adequacy of the sample was confirmed by identifying pancreatic acini on a wet preparation. In the case of dual pancreas and kidney biopsy, the kidney biopsy was also performed under computed tomography guidance. In the case of kidney biopsy alone, kidney biopsies were performed using real-time ultrasound guidance and a coaxial needle introducer. Two to 3 cores were usually collected with an 18-gauge biopsy gun, and sample adequacy was assessed by visualizing glomeruli on the surface of the retrieved tissue. Both kidney and pancreas biopsy specimens were evaluated and scored according to the published Banff criteria.^19,20^

### Variables

The primary variables analyzed included dd-cfDNA level, BANFF classification of kidney and pancreas biopsies, laboratory markers (lipase, creatinine), and time intervals between dd-cfDNA testing and biopsy.

### Statistical Analysis

Descriptive statistics for each variable are reported. A significance level (alpha) of 0.05 was specific for 2-tailed tests. Comparative analysis included chi-square or Fisher exact tests for discrete variables and Student *t* test or Mann-Whitney *U* test for continuous variables. The diagnostic accuracy of dd-cfDNA for detecting allograft rejection was assessed using standard statistical measures. Sensitivity was defined as the proportion of true positive cases (biopsy-proven rejection with high-risk dd-cfDNA) among all actual rejection cases. Specificity was defined as the proportion of true negative cases (no rejection on biopsy with low-risk dd-cfDNA) among all nonrejection cases. The positive predictive value (PPV) was defined as the proportion of positive dd-cfDNA results that were true positives while the negative predictive value (NPV) was defined as the proportion of negative dd-cfDNA results that were true negatives. Statistical analysis was conducted with GraphPad Prism (Boston, MA). The interquartile range is reported as a single value, calculated as Q3 minus Q1, representing the difference between the 75th and 25th percentiles.

### Borderline Acute Cellular Rejection

For the purposes of calculating sensitivity, specificity, PPV, and NPV, borderline acute cellular rejection (ACR) was considered negative for rejection.

## RESULTS

### Baseline Characteristics

Forty-two pancreas transplant recipients were included in this study, comprising 38 SPK (90.5%) and 4 PTA (9.5%) recipients. Twenty-two of 42 (52.4%) recipients were male, and median age at time of pancreas transplant was 48 yrs. A total of 50 dd-cfDNA tests and 45 adequate organ biopsies were included in this analysis. Median (interquartile range) time from pancreas transplant to organ biopsy was 4 mo (16 mo) and ranged from 0 to 126 mo. Thirty-three of 42 (78.6%) recipients underwent dd-cfDNA testing before or the same day as biopsy, while 9 of 42 (21.4%) recipients underwent dd-cfDNA testing after biopsy. The time difference between dd-cfDNA testing and organ biopsy ranged from 25 d before biopsy to 29 d after biopsy.

### dd-cfDNA Testing in SPK Recipients

A total of 36 SPK recipients underwent organ biopsy with concurrent dd-cfDNA testing performed either pre-biopsy or before antirejection treatment initiation (Table 1). Additional donor and recipient clinical details are provided in **Tables S1** and **S2** (**SDC**, https://links.lww.com/TXD/A850). Fourteen recipients (38.9%) had low-risk dd-cfDNA results, while 22 (61.1%) exhibited high-risk dd-cfDNA levels. Among the low-risk cohort, 12 of 14 biopsies (85.7%) were negative for rejection (true negatives), whereas 2 of 14 (14.3%) demonstrated rejection (false negatives). In the high-risk group, patient number 1 had a pancreas biopsy that yielded inadequate tissue for diagnostic interpretation, resulting in a total of 24 dd-cfDNA/adequate biopsy results for analysis (including 3 patients who had 2 sequential tests). Of these 24 tests, 13 of 24 (54.2%) biopsies confirmed rejection (true positives) and 11 of 24 (45.8%) were negative for rejection (false positives). Using these data, dd-cfDNA testing in this cohort demonstrated a sensitivity of 86.7% (13/15), specificity of 52.2% (12/23), PPV of 54.2% (13/24), and NPV of 85.7% (12/14). One biopsy was indeterminate and 1 inadequate, both were excluded from performance calculations.

**TABLE 1.** SPK recipients with low-risk dd-cfDNA (N = 14) and high-risk dd-cfDNA (N = 22)

Recipient	dd-cfDNA result (%)	Days from dd-cfDNA to biopsy	Months from transplant to biopsy^a^	Biopsy^b^		Other injuries	Indication for biopsy
				Pancreas	Kidney		
Low-risk dd-cfDNA							
1	Low risk (0.1)	–17	126	–	ACR (borderline)	URI	↑ Lipase and creatinine
2	Low risk (0.2)	–14	5	Negative	–	None	↑ Lipase and creatinine
3	Low risk (0.7)	–12	4	–	Negative	BK nephropathy	↑ Creatinine
4	Low risk (0.3)	–10	3	–	Negative	None	↑ Creatinine
5	Low risk (0.1)	–5	14	Negative	–	None	↑ Lipase and creatinine
6	Low risk (0.8)	–4	39	–	Negative	None	Subtherapeutic IS levels
7	Low risk (0.2)	–2	3	–	Negative	Abscess	Fevers
8	Low risk (0.4)	–1	1	ACR (grade 1)	–	Diarrhea	↑ Lipase
9	Low risk (0.7)	–1	9	Negative	–	BK viremia	↑ Lipase and creatinine
10	Low risk (0.9)	0	16	–	Negative	Pyelonephritis	↑ Lipase and creatinine
11	Low risk (0.4)	0	35	AMR	–	None	↑ Lipase
12	Low risk (0.1)	0	4	–	ACR (borderline)	None	↑ Lipase and creatinine
13	Low risk (0.2)	1	64	Negative	–	None	↑ Lipase and creatinine
14	Low risk (0.6)	2	0	–	Negative	FUO	↑ Lipase and creatinine
High-risk dd-cfDNA							
1	High risk (1.7)	0	72	Inadequate			↑ Lipase and creatinine
2	High risk (1.1)	0	1	–	Mixed	None	
3	High risk (1.1)	–25	2	ACR (grade 1)	–	None	↑ Lipase
4	High risk (5.7)	–23	58	–	Negative	None	↑ Lipase and creatinine
5	High risk (6.6)	–23	107	ACR (grade 2)	Mixed	None	↑ Lipase and creatinine
6	High risk (1.6)	–20	13	ACR (grade 1)	–	None	↑ Lipase
	High risk (1.4)	–14	2	AMR	–	None	↑ Lipase
7	High risk (7.5)	–7	2	–	ACR (grade 1)	Pancreatitis	↑ Lipase
8	High risk (2.3)	–7	1	–	Negative	None	↑ Lipase
9	High risk (3.1)	–6	1	–	Negative	FUO/FUO/TMA	FUO
	High risk (3.1)	–3	1	Negative	–		FUO
10	High risk (1.9)	–6	7	–	Mixed	CMV colitis	↑ Lipase and creatinine
11	High risk (5.8)	–4	2	–	ACR (borderline)	Pancreatic leak	↑ Lipase and creatinine
12	High risk (6.9)	–4	1	–	Mixed	GI infection	↑ Lipase and creatinine
13	High risk (5.8)	–4	1	Negative	–	Pancreatitis	FUO
	High risk (5.8)	–2	1	–	Negative		FUO
14	High risk (3.1)	–4	1	–	Negative	FUO	FUO
15	High risk (1.2)	–4	1	–	Negative	None	↑ Lipase and creatinine
16	High risk (2.4)	–3	12	ACR (grades 1–2)	–	None	↑ Lipase
17	High risk (1.7)	–1	40	ACR (grade 3)	–	None	↑ Lipase and creatinine
18	High risk (8.7)	–1	11	ACR (grade 1)	–	CMV viremia	↑ Lipase
19	High risk (3.7)	–1	1	–	Negative	GI infection	↑ Creatinine
20	High risk (4.1)	0	1	Indeterminate	–	GI infection	↑ Creatinine, GI infection
21	High risk (22.5)	0	32	AMR	–	None	↑ Lipase and creatinine
22	High risk (4.9)	3	1	–	Negative	TMA	FUO

^*a*^Months from transplant to biopsy—values correspond to 30-d intervals: 0 = 0–30 d posttransplant; 1 = 31–60 d; 2 = 61–90 d; 3 = 91–120 d; and so on.

^*b*^For the purposes of calculating sensitivity, specificity, PPV, and NPV, borderline ACR was considered negative for rejection.

ACR, acute cellular rejection; AMR, antibody-mediated rejection; CMV, cytomegalovirus; dd-cfDNA, donor-derived cell-free DNA; FUO, fever of unknown origin; GI, gastrointestinal; IS, immunosuppression; NPV, negative predictive value; PPV, positive predictive value; SPK, simultaneous pancreas kidney; TMA, thrombotic microangiopathy; URI, upper respiratory infection; –, not performed.

Similarly, in SPK recipients with low-risk dd-cfDNA undergoing pancreas biopsy, 2 of 6 (33.3%) exhibited rejection and 4 of 6 (66.7%) demonstrated no rejection (Table 1). Among 11 adequate pancreas biopsies with high-risk dd-cfDNA results, 8 exhibited rejection, 2 were negative for rejection, and 1 was indeterminate (excluded from analysis). For pancreas biopsies, dd-cfDNA demonstrated a sensitivity of 80.0%, specificity of 66.7%, PPV of 80.0%, and NPV of 66.7%.

Several other injury diagnoses were identified among high-risk dd-cfDNA SPK recipients with negative biopsies, potentially accounting for false positive results in this cohort. These diagnoses were temporally associated with dd-cfDNA sampling and biopsy, occurring within days, rather than remote diagnoses. Specifically, thrombotic microangiopathy (n = 2), pancreatitis (n = 1), pancreatic leak (n = 1), gastrointestinal infection (n = 1), and fever of unknown origin (n = 1) were documented as alternative sources of graft injury.

### dd-cfDNA Accuracy Beyond the Early Perioperative Period

Given prior studies suggesting that dd-cfDNA elevation within the first 45 d posttransplant may reflect ischemia-reperfusion injury rather than immunologic rejection, we performed a subset analysis excluding all biopsies obtained within the first 60 d posttransplant (corresponding to months 0–1 in Table 1). After excluding these early biopsies, dd-cfDNA demonstrated improved diagnostic accuracy. Among the retained cohort, 10 of 12 high-risk tests corresponded to biopsy-proven rejection (true positives = 10; false positives = 2), and 11 of 12 low-risk tests were negative for rejection (true negatives = 11; false negatives = 1). The resulting test characteristics were sensitivity 90.9%, specificity 84.6%, PPV 83.3%, and NPV 91.7%.

### dd-cfDNA Testing in PTA Recipients

Four PTA recipients underwent dd-cfDNA testing and concurrent biopsy (Table 2). Additional donor and recipient clinical details are provided in **Table S3** (**SDC**, https://links.lww.com/TXD/A850). One recipient underwent 2 separate rounds of dd-cfDNA testing and biopsy 7 mo apart. In total, there were 2 high-risk and 3 low-risk dd-cfDNA results. Both high-risk results corresponded to biopsies that revealed ACR, while all 3 low-risk results were associated with biopsies negative for rejection. Based on these findings, the sensitivity and specificity of dd-cfDNA for detecting rejection in this cohort were both 100%. The PPV and NPV were also 100%, as all high-risk results correctly identified rejection and all low-risk results correctly excluded it.

**TABLE 2.** PTA recipients (N = 4)

Recipient	dd-cfDNA result (%)	Days from dd-cfDNA to biopsy	Months from transplant to biopsy^a^	Biopsy^b^	
				Pancreas	Kidney
1	High risk (2.9)	–4	11	ACR (grades 1–2)	–
	Low risk (0.2)	–9	4	Negative	–
2	High risk (1.4)	1	1	ACR (grade 1)	–
3	Low risk (0.1)	0	29	Negative	–
4	Low risk (0.1)	29	3	Negative	–

^*a*^Months from transplant to biopsy—values correspond to 30-d intervals: 0 = 0–30 d posttransplant; 1 = 31–60 d; 2 = 61–90 d; 3 = 91–120 d; and so on.

^*b*^For the purposes of calculating sensitivity, specificity, PPV, and NPV, borderline ACR was considered negative for rejection.

ACR, acute cellular rejection; dd-cfDNA, donor-derived cell-free DNA; NPV, negative predictive value; PPV, positive predictive value; PTA, pancreas transplant alone; –, not performed.

When combining the SPK and PTA recipient cohorts, dd-cfDNA testing demonstrated an overall sensitivity of 88.2% (15/17), specificity of 57.7% (15/26), PPV of 57.7% (15/26), and NPV of 88.2% (15/17) for detecting rejection (**Table S4**, **SDC**, https://links.lww.com/TXD/A850).

We then evaluated the diagnostic performance of dd-cfDNA by individual organ in SPK recipients (Table 1). Among those with low-risk dd-cfDNA undergoing kidney biopsy, 0 of 8 biopsies exhibited rejection (false negatives), while all 8 demonstrated no rejection (true negatives). For high-risk dd-cfDNA and kidney biopsy, 5 of 14 biopsies (35.7%) showed rejection (true positives), while 9 of 14 (64.3%) were negative for rejection (false positives). This yielded a sensitivity of 100% (5/5), specificity of 47.1% (8/17), PPV of 35.7% (5/14), and NPV of 100% (8/8) when evaluating dd-cfDNA to kidney histology only.

When evaluating the diagnostic performance of dd-cfDNA to detect pancreas rejection (SPK and PTA combined), dd-cfDNA demonstrated excellent diagnostic performance. Among the combined cohort, 4 of 9 cases with low-risk dd-cfDNA exhibited no evidence of rejection (true negatives), while 5 of 9 had biopsy-proven rejection (false negatives). For high-risk dd-cfDNA, 10 of 13 biopsies were positive for rejection (true positives), and 3 were negative or indeterminate for rejection (false positives/indeterminate excluded). This corresponds to a sensitivity of 83.3% (10/12), specificity of 66.7% (4/6), PPV of 76.9% (10/13), and NPV of 80% (4/5) across both SPK and PTA pancreas biopsy cohorts combined.

### Quantitative dd-cfDNA Levels Across Rejection Types and Transplant Categories

**FIGURE 1.**
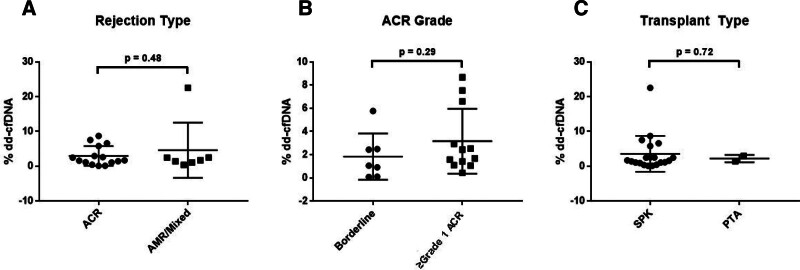
Quantitative dd-cfDNA% values across rejection and transplant categories (N = 22). Comparative visualization of 22 organ biopsies with confirmed rejection and the corresponding dd-cfDNA% values. A, ACR vs AMR/mixed rejection. B, Borderline vs ≥grade 1 ACR in those with ACR. C, SPK vs PTA. ACR, acute cellular rejection; AMR, antibody-mediated rejection; dd-cfDNA, donor-derived cell-free DNA; PTA, pancreas transplant alone; SPK, simultaneous pancreas kidney.

Next, we investigated whether quantitative differences in dd-cfDNA levels existed across rejection type, cellular rejection grade, or transplant type (Figure 1). Among 24 organ biopsies with confirmed rejection, 22 had corresponding dd-cfDNA testing performed either pre-biopsy or before antirejection treatment initiation. Comparative analysis revealed no significant quantitative differences in dd-cfDNA levels to distinguish between rejection types (ACR versus AMR/mixed rejection; *P* = 0.48), grades of cellular rejection (borderline versus ≥grade 1 ACR; *P* = 0.29), or transplant categories (SPK versus PTA; *P* = 0.72; Figure 1A–C).

### Dd-cfDNA% in Subclinical Rejection

Next, the accuracy of dd-cfDNA in detecting subclinical rejection, defined as rejection occurring in the absence of laboratory elevations in either creatinine or lipase, was assessed. A total of 47 organ biopsies from 40 pancreas transplant recipients were analyzed. All dd-cfDNA testing was performed before biopsy or before the initiation of antirejection therapy, if applicable. Of the 47 biopsies, 13 were conducted without concurrent laboratory evidence of graft dysfunction (Table 3). Among these, rejection was confirmed in 2 cases, each of which was associated with a high-risk dd-cfDNA result. Of the remaining 11 biopsies without rejection, 6 (54.5%) were associated with an increased- and 5 (45.5%) with a low-risk dd-cfDNA result. This corresponds to a sensitivity of 100% (2/2), specificity of 45.5% (5/11), PPV of 25.0% (2/8), and NPV of 100% (5/5). dd-cfDNA results did not appear to correlate with near term development of de novo donor-specific antibody.

**TABLE 3.** dd-cfDNA% in subclinical rejection

Recipient^a^	Transplant	dd-cfDNA result (%)	Days from dd-cfDNA to biopsy	Months from transplant to biopsy^b^	Biopsy^c^		De novo DSA	Indication for dd-cfDNA testing
					Pancreas	Kidney		
1	SPK	High risk (1.1)	0	1	–	Mixed	Yes	De novo DSA
2	SPK	High risk (6.9)	–4	1	–	Mixed	No	FUO
3	SPK	High risk (3.1)	–6	1	–	Negative	No	FUO
		High risk (3.1)	–3	1	Negative	–	No	FUO
4	SPK	High risk (5.8)	–2	1	–	Negative	No	FUO
		High risk (5.8)	–4	1	Negative	–	No	FUO
5	SPK	High risk (3.1)	–4	1	–	Negative	Yes	De novo DSA
6	SPK	High risk (4.9)	3	1	–	Negative	Yes	De novo DSA
7	SPK	Low risk (0.2)	5	2	–	Negative	Yes	De novo DSA
8	SPK	Low risk (0.2)	–2	3	–	Negative	No	FUO
9	SPK	Low risk (0.8)	–4	39	–	Negative	Yes	De novo DSA
10	PTA	Low risk (0.1)	0	29	Negative	–	No	Sub-IS
11	PTA	Low risk (0.2)	–9	4	Negative	–	Yes	De novo DSA

Thirteen biopsies undertaken in the absence of biochemical elevations (creatinine or lipase). These are still considered “for cause” biopsies as the indication for biopsy included FUO, new de novo DSA, or subtherpeutic immunosuppression levels.

^*a*^All recipients from Table 3 are included in either Table 1 or Table 2.

^*b*^Months from transplant to biopsy—values correspond to 30-d intervals: 0 = 0–30 d posttransplant; 1 = 31–60 d; 2 = 61–90 d; 3 = 91–120 d; and so on.

^*c*^For the purposes of calculating sensitivity, specificity, PPV, and NPV, borderline ACR was considered negative for rejection.

dd-cfDNA, donor-derived cell-free DNA; DSA, donor-specific antibody; FUO, fever of unknown origin; NPV, negative predictive value; PPV, positive predictive value; PTA, pancreas transplant alone; SPK, simultaneous pancreas-kidney; Sub-IS, subtherapeutic immunosuppression; –, not performed.

### Impact of Rejection Treatment on dd-cfDNA%

Three recipients (2 SPK, 1 PTA) with initial high-risk dd-cfDNA results and biopsy-confirmed rejection underwent posttreatment surveillance dd-cfDNA testing (Table 4). All 3 demonstrated normalization of organ-specific laboratory markers (lipase/creatinine) following treatment of rejection. Two recipients transitioned from high-risk to low-risk dd-cfDNA levels after treatment and required no further intervention. In contrast, 1 recipient (recipient 3) maintained high-risk dd-cfDNA levels, albeit quantitatively reduced from 8.7% pretreatment to 2.8% posttreatment, despite normalized laboratory values. This discordance prompted a repeat biopsy, which revealed persistent subclinical rejection (ACR grade 1), leading to an additional treatment course. An additional 2 patients (recipients 4 and 5—both SPK) underwent dd-cfDNA testing following completion of treatment for biopsy-confirmed rejection, although neither had pretreatment dd-cfDNA testing. dd-cfDNA testing were low-risk in both recipients, which coincided with normalization of clinical laboratory values. Neither patient received additional treatment.

**TABLE 4.** Impact of rejection treatment on dd-cfDNA%

Recipient	Transplant	dd-cfDNA result pretreatment (%)	Days from dd-cfDNA to biopsy	Months from transplant to biopsy^a^	Biopsy		dd-cfDNA result posttreatment (%)
					Pancreas	Kidney	
1^b^	SPK	High risk (6.9)	–4	1	–	Mixed	Low risk (0.2)
2^c^	PTA	High risk (1.4)	1	1	ACR (grade 1)	–	Low risk (0.1)
3^d^^,e^	SPK	High risk (8.7)	–1	11	ACR (grade 1)	–	High risk (2.8)
4^f^	SPK	Not performed	19	19	ACR (grade 1)	–	Low risk (0.2)
5^f^	SPK	Not performed	19	4	ACR (grade 1)	–	Low risk (0.2)

^*a*^Months from transplant to biopsy—values correspond to 30-d intervals: 0 = 0–30 d posttransplant; 1 = 31–60 d; 2 = 61–90 d; 3 = 91–120 d; and so on.

^*b*^Recipient 1 in this table = recipient 12 from Table 1 (high risk), with an additional dd-cfDNA result posttreatment presented in this table.

^*c*^Recipient 2 in this table = recipient 2 from Table 2, with an additional dd-cfDNA result posttreatment presented in this table.

^*d*^Recipient 3 in this table—recipient 18 from Table 1 (high risk), with an additional dd-cfDNA result posttreatment presented in this table.

^*e*^Recipient 3 underwent repeat biopsy because of persistently elevated post treatment dd-cfDNA% levels, which demonstrated ongoing rejection.

^*f*^Recipients 4 and 5 in this table are new recipients with only dd-cfDNA posttreatment results.

ACR, acute cellular rejection; dd-cfDNA, donor-derived cell-free DNA; PTA, pancreas transplant alone; SPK, simultaneous pancreas-kidney; –, not performed.

## DISCUSSION

Although dd-cfDNA has emerged as a promising noninvasive biomarker in solid organ transplantation, its application in pancreas transplant recipients—including those with multiorgan transplants—remains underexplored. In this study, we provide 1 of the largest single-center analyses to date assessing the diagnostic performance of dd-cfDNA for detecting rejection in both SPK and PTA recipients. Our findings highlight that dd-cfDNA offers considerable utility as a surveillance tool for ruling out rejection in SPK recipients, reflecting its high sensitivity and NPV. Nevertheless, positive dd-cfDNA results must be interpreted within the broader clinical context and confirmed with additional diagnostic methods such as biopsy, given their limited PPV and specificity. As such, dd-cfDNA should augment, rather than replace, traditional clinical and histological monitoring strategies for pancreas transplant recipients.

Previous studies evaluating dd-cfDNA in pancreas transplantation have provided important early insights.^15,16^ In one of the first prospective studies of dd-cfDNA in pancreas transplant recipients, Ventura-Aguiar et al^16^ demonstrated that elevated dd-cfDNA levels were associated with biopsy-proven rejection; however, their cohort was limited to SPK recipients, of which the vast majority (68%) underwent protocol biopsy rather than for-cause biopsy. A study by Yoo et al^15^ included a larger cohort and incorporated both SPK and PTA recipients, but primarily reported on cross-sectional dd-cfDNA measurements and did not directly compare diagnostic performance by organ or risk stratification thresholds. In contrast, the current study expands upon these findings by analyzing a larger, single-center cohort of SPK and PTA recipients, and systematically correlating dd-cfDNA results with concurrent biopsy findings to assess test characteristics—including sensitivity, specificity, PPV, and NPV—for each type of transplant recipient. Additionally, our study uniquely evaluates the utility of dd-cfDNA in detecting subclinical rejection and demonstrates the ability of dd-cfDNA to detect both pancreas and kidney allograft rejection with good sensitivity.

In our combined cohort of pancreas transplant recipients, dd-cfDNA testing for pancreas transplant rejection demonstrated a sensitivity of 88.2%, specificity of 55.6%, PPV of 55.6%, and NPV of 88.2. In the kidney transplant literature, dd-cfDNA has demonstrated sensitivities between 71% and 74% and specificities as high as 92%–93% at thresholds of 0.5%–1%.^11,21^ The lower specificity and PPV observed in our study may be attributed to the increased incidence of nonrejection graft injury and the presence of other injuries, which are more common in pancreas transplant recipients and can elevate dd-cfDNA independent of rejection. Despite this, our NPV remains high and comparable to other studies, underscoring dd-cfDNA’s clinical value as a noninvasive rule-out test for rejection. The moderate specificity in our cohort highlights the importance of integrating dd-cfDNA results with clinical and histopathologic findings, particularly in the context of multiorgan transplantation and complex posttransplant courses.

Although limited by a small sample size, our results suggest that dd-cfDNA may serve as a valuable surveillance tool following treatment for rejection in pancreas transplant recipients, potentially reducing reliance on posttreatment “closure” biopsies. The dd-cfDNA levels observed over the course of this study declined in response to effective antirejection therapy, corroborating findings from other recent studies that demonstrated dd-cfDNA trajectories can reflect ongoing graft injury or recovery.^22^ This dynamic surveillance capability offers a noninvasive alternative to repeat biopsy, allowing clinicians to track allograft status in real time and identify persistent or recurrent rejection earlier than traditional biochemical markers. While dd-cfDNA has demonstrated value as a posttreatment surveillance tool, decisions regarding escalation of immunosuppression should not be based solely on elevated dd-cfDNA results. Integrating dd-cfDNA measurements with clinical parameters and histological confirmation remains critical to reliably guide posttransplant management, particularly in previously treated recipients.

The observed heterogeneity in dd-cfDNA testing and biopsy coordination in our study reflects the natural effect of an initial, real-world experience during a period when no standardized protocol existed for pancreas transplant recipients. Decisions regarding dd-cfDNA testing and biopsy timing were made based on individual clinical scenarios, often in response to signs of graft dysfunction or suspicion for rejection, and in some cases, dd-cfDNA was used to monitor response following treatment. Recognizing the need for a more systematic approach, our center has since developed and implemented a protocol based on the data presented in this report accumulated over the past 4 y. dd-cfDNA is now ordered at the initial indication of immune-mediated injury, such as the emergence of de novo DSA, new insulin needs, or laboratory abnormalities (elevated lipase or creatinine above baseline), but is not used at predetermined posttransplant time points (ie, protocol draws). Additionally, dd-cfDNA is used to monitor treatment response in cases of high-grade rejection (Banff grade 2 or higher). This protocol should provide more consistency in testing and patient management in pancreas transplant recipient.

Some limitations of this study should be noted, and include the following: First, the retrospective, single-center design may have introduced selection bias and could limit the generalizability of the findings. Second, the small sample size reduces statistical power and may increase the potential for overinterpretation of results, particularly for subgroup analyses and for distinguishing between different rejection phenotypes. Next, the decision to perform a for-cause biopsy was at the discretion of the treating clinician, which may have introduced additional bias, and the timing of dd-cfDNA sampling in relation to clinical events and interventions was not standardized, potentially impacting dd-cfDNA levels differently across patients. It is also important to note that, currently, clinically available dd-cfDNA assays do not provide organ-specific results in multiorgan transplant recipients (such as SPK) and do not distinguish between different types of rejection (cellular versus antibody-mediated). Finally, future studies incorporating simultaneous biopsy of both kidney and pancreas would be valuable in clarifying the concordance between dd-cfDNA levels and organ-specific rejection in SPK recipients.

## CONCLUSIONS

In summary, this study demonstrates that dd-cfDNA is a promising noninvasive biomarker for detecting and monitoring rejection in pancreas transplant recipients. Furthermore, our findings suggest that dd-cfDNA can enhance posttransplant surveillance and may reduce reliance on invasive biopsies. Future prospective, multicenter studies are warranted to further validate these results and to optimize the integration of dd-cfDNA testing into clinical practice.

## Supplementary Material


