# Supplementary material for: Donor-derived Cell-free DNA for Detection of Rejection After Pancreas Transplantation
Source: Transplant Direct. 2026 Apr 8;12(5):e1939. doi: 10.1097/TXD.0000000000001939 (PMC13065230; doi:10.1097/TXD.0000000000001939)
Supplement: Supplementary file 1 [file txd-12-e1939-s001.pdf]

Patient #	Donor Age at Transplant (years)	Donor BMI (kg/m^2)	Recipient CMV Status	Donor CMV Status	Recipient cPRA (%)
1	21.4	22.8	Positive	Positive	0.0
2	23.7	26.7	Negative	Negative	62.0
3	28	27.2	Positive	Positive	52.0
4	37	26.2	Positive	Positive	35.0
5	17.3	20.6	Positive	Positive	55.0
6	30	29.3	Negative	Positive	7.0
7	31	22.1	Positive	Positive	84.0
8	29	21.6	Positive	Negative	26.0
9	17	17.9	Positive	Positive	59.0
10	7	20.2	Positive	Negative	43.0
11	35.1	31.7	Positive	Positive	17.0
12	27	27.3	Positive	Positive	24.0
13	31.1	22.9	Negative	Negative	—
14	33	18.3	Positive	Positive	37.0

Supplementary Table 1. Baseline donor and recipient characteristics for simultaneous pancreas–kidney (SPK) recipients with low-risk dd-cfDNA results (<1%) at the time of biopsy, including donor age, donor body mass index (BMI), donor and recipient

cytomegalovirus (CMV) serostatus, and recipient calculated panel reactive antibody (cPRA) at transplant.

**Patient #**	**Donor Age at Transplant (years)**	**Donor BMI (kg/m^2)**	**Donor CMV Status**	**Recipient CMV Status**	**Recipient cPRA (%)**
1	22.5	—	Negative	Positive	10
2	13	26.7	Positive	Negative	88
3	29	30.6	Positive	Negative	8
4	25	19.7	Negative	Negative	6
5	16.2	22.2	Positive	Positive	65
6	27	23.8	Negative	Positive	96
7	17	23	Negative	Negative	3
8	27	23.1	Positive	Negative	95
9	14	14.3	Negative	Negative	54
10	20	23.8	Positive	Negative	23
11	28	21.8	Negative	Positive	11
12	14	19.8	Negative	Positive	0
13	19	20.7	Positive	Positive	16

Patient #	Donor Age at Transplant (years)	Donor BMI (kg/m^2)	Donor CMV Status	Recipient CMV Status	Recipient cPRA (%)
14	30	21	Negative	Negative	0
15	31	25	Negative	Positive	96
16	9.5	21.4	Negative	Positive	0
17	22	19.8	Positive	Negative	28
18	24	24.8	Positive	Negative	0
19	27	25	Positive	Negative	0
20	37	28.1	Negative	Positive	0
21	27.2	23.9	Positive	Positive	0
22	25	19.8	Positive	Negative	3

Supplementary Table 2. Baseline donor and recipient characteristics for SPK recipients with high-risk dd-cfDNA results (≥1%) at the time of biopsy, including donor age, donor BMI, donor and recipient CMV serostatus, and recipient cPRA at transplant.

Patient #	Donor Age at Transplant (years)	Donor BMI (kg/m^2)	Recipient CMV Status	Donor CMV Status	Recipient cPRA (%)
1	22	24.6	Positive	Positive	3
2	10	28.6	Positive	Negative	99
3	21.5	19.8	Negative	Positive	--
4	20	19.3	Positive	Negative	100

Supplementary Table 3. Baseline donor and recipient characteristics for pancreas transplant alone (PTA) recipients who underwent for-cause biopsy with contemporaneous dd-cfDNA testing, including donor age, donor BMI, recipient and donor CMV serostatus, and recipient cPRA at transplant.

Metric	SPK	PTA	Combined Cohort
Sensitivity	86.7%	100%	88.2%
Specificity	50.0%	100%	55.6%
PPV	52.0%	100%	55.6%
NPV	85.7%	100%	88.2%

Supplemental Table 4. SPK, Simultaneous Pancreas Kidney; PTA, Pancreas Transplant Alone; PPV, positive predictive value; NPV, negative predictive value
